# Testin protects against cardiac hypertrophy by targeting a calcineurin‐dependent signalling pathway

**DOI:** 10.1111/jcmm.13934

**Published:** 2018-11-22

**Authors:** Lu Gao, Yuan Liu, Sen Guo, Lili Xiao, Cui Liang, Xiaofang Wang

**Affiliations:** ^1^ Department of Cardiology The First Affiliated Hospital of Zhengzhou University Zhengzhou China

**Keywords:** calcineurin, cardiac hypertrophy, signalling pathway, testin

## Abstract

Multiple organs express testin (TES), including the heart. Nevertheless, current understanding of the influence of TES on cardiovascular diseases, especially on cardiac hypertrophy and its etiology, is insufficient. This study investigated the influence of TES on cardiac hypertrophy and its etiology. Murine models with excessive TES expression specific to the heart were constructed with an adeno‐associated virus expression system. Cardiac hypertrophy was stimulated through aortic banding (AB). The severity of cardiac hypertrophy was evaluated through molecular, echocardiographic, pathological, and hemodynamic examination. The findings of our study revealed that TES expression was remarkably suppressed not only in failing human hearts but also in mouse hearts with cardiac hypertrophy. It was discovered that excessive TES expression driven by an adeno‐associated viral vector noticeably inhibited hypertrophy triggered by angiotensin II (Ang II) in cultivated cardiomyocytes from newborn rats. It was also revealed that TES knockdown via AdshTES caused the reverse phenotype in cardiomyocytes. Furthermore, it was proved that excessive TES expression attenuated the ventricular dilation, cardiac hypertrophy, dysfunction, and fibrosis triggered by AB in mice. It was discovered that TES directly interacted with calcineurin and suppressed its downstream signalling pathway. Moreover, the inactivation of calcineurin with cyclosporin A greatly offset the exacerbated hypertrophic response triggered by AB in TES knockdown mice. Overall, the findings of our study suggest that TES serves as a crucial regulator of the hypertrophic reaction by hindering the calcineurin‐dependent pathway in the heart.

## 1 INTRODUCTION

Characterized as an enlargement of the myocardial mass, cardiac hypertrophy is a remodelling of the myocardium in response to multiple intrinsic and extrinsic irritants.^1^, ^2^ Despite the fact that the hypertrophic reaction compensates for an elevation in workload, continuous cardiac hypertrophy brings about congestive heart failure, arrhythmia, and ultimately sudden death.^3^, ^4^, ^5^ Consequently, determining how to inhibit cardiac hypertrophy is an urgent challenge to attenuate the end‐organ injury caused by pressure overload. Elucidating the etiology of cardiac hypertrophy and its progression to congestive heart failure is necessary.

The human testin (TES) gene was originally identified in 1995 and was noted to have its highest expression in the testes, yet it is also widely expressed throughout other tissues.^6^, ^7^ In humans, the TES gene is located in the FRG7G region on chromosome 7q31.2, which is believed to contain a tumour suppressor gene because of its lack of heterozygosity in multiple malignancies.^8^, ^9^ The interest in TES mainly arises from the fact that it has been shown to be downregulated in an increasing number of human tumour types, in which its downregulation correlates with disease progression.^10^, ^11^, ^12^, ^13^, ^14^

As a modular scaffold protein, TES contains a central PET (Prickle, Espinas, Testin) domain, a cysteine‐rich region, and three LIM domains that compose the C‐terminal half of TES.^14^, ^15^, ^16^ The LIM domain was initially discovered in three transcription factors essential to development, Isl‐1, Mec‐3, and Lin‐11.^17^, ^18^ Later, various proteins with LIM domains were recognized that participate in various biological processes such as differentiation, the generation of malignancies, and the organization of the cytoskeleton.^19^, ^20^, ^21^ The LIM domain of TES resembles that of other proteins, including FLH2, CRP3, and cysteine‐rich domains 1 (Lmcd1), which serve as crucial modulators of cardiovascular diseases, especially cardiac hypertrophy, suggesting the potential participation of TES in cardiac hypertrophy.^22^, ^23^, ^24^ Consequently, we hypothesized that TES could be a promising modulator of cardiac hypertrophy.

It was discovered in this study that the concentration of TES was suppressed in the hearts of patients suffering from dilated cardiomyopathy (DCM) as well as in cardiac hypertrophy models triggered by pressure overload. With the help of an adeno‐associated virus expression system, it was discovered that pressure overload‐stimulated cardiac hypertrophy was suppressed in mice with excessive TES expression. TES was able to directly interact with calcineurin and inhibit downstream reactions. Furthermore, calcineurin deactivation by cyclosporin A (CsA) clearly offset the amplified hypertrophic reaction stimulated by pressure overload in TES knockdown mice. Our research suggests that TES is essential in the regulation of cardiac hypertrophy and can be used as a reliable target to treat cardiac hypertrophy.

## 2 MATERIALS AND METHODS

### 2.1 Reagents

Ang II and CsA were acquired from Sigma‐Aldrich (St. Louis, MO, USA). A bicinchoninic acid (BCA) protein assay kit was acquired from Pierce (Rockford, IL, USA). Secondary antibodies conjugated with peroxidase (Jackson ImmunoResearch Laboratories, West Grove, PA, USA) were applied to observe the binding of the primary antibody (Table 1). Fetal calf serum was procured from HyClone (Waltham, MA, USA). Cell cultivation reagents and other reagents were provided by Sigma‐Aldrich.

**Table 1:** Primer sequences used for RT‐PCR

mRNA	Forward	Reverse
TES^a^	TGGTCAAGAAGAGCATGACG	GCCACTGGATTGGTCAAGAT
TES^b^	ACAGGCTTTCAAAGGCAGAA	ATCCCATGCAGTCCAGTAGG
TES^c^	CCCTTCAAAGTGCCATGAGT	TGTGCTCAGCAGATTTGTCC
GAPDH^c^	CATCACCATCTTCCAGGAGCGAGA	TGCAGGAGGCATTGCTGATGATCT
ANP^a^	ACCTGCTAGACCACCTGGAG	CCTTGGCTGTTATCTTCGGTACCGG
BNP^a^	GAGGTCACTCCTATCCTCTGG	GCCATTTCCTCCGACTTTTCTC
β‐MHC^a^	CCGAGTCCCAGGTCAACAA	CTTCACGGGCACCCTTGGA
Col1agenI^a^	AGGCTTCAGTGGTTTGGATG	CACCAACAGCACCATCGTTA
Col1agenIII^a^	AAGGCTGCAAGATGGATGCT	GTGCTTACGTGGGACAGTCA
CTGF^a^	AGGGCCTCTTCTGCGATTTC	CTTTGGAAGGACTCACCGCT
GAPDH^a^	ACTCCACTCACGGCAAATTC	TCTCCATGGTGGTGAAGACA
ANP^b^	AAAGCAAACTGAGGGCTCTGCTCG	TTCGGTACCGGAAGCTGTTG CA
β‐MHC^b^	TCTGGACAGCTCCCCATTCT	CAAGGCTAACCTGGAGAAGATG
GAPDH b	GACATGCCGCCTGGAGAAAC	AGCCCAGGATGCCCTTTAGT

Sequences are listed 5′–3′.

a The PCR used the primers in mice.

b The PCR used the primers in neonatal rat cardiomyocytes.

c The PCR used the primers in human.

### 2.2 Human heart specimens

Samples of failing hearts were acquired from the left ventricles (LVs) of patients who suffered from DCM and had received treatments subsequent to heart transplantation (Table 2). Control samples were acquired from the LVs of normal donors. Written informed consent was obtained from the families of the prospective heart donors. This study complied with a protocol approved by the First Affiliated Hospital of Zhengzhou University Human Research Ethics Committee, and samples were collected after informed consent.

**Table 2:** Information of human DCM hearts and donor hearts

n	Type	Age	Sex	Cardiac function		Therapy before transplantation			
				LVEF (%)	LVEDD(mm)	ACEI	Beta‐blocker	Diuretics	Digoxin
1	Donor	37	F	N/A	N/A	N/A	N/A	N/A	N/A
2	Donor	30	F	N/A	N/A	N/A	N/A	N/A	N/A
3	Donor	28	M	N/A	N/A	N/A	N/A	N/A	N/A
4	Donor	43	M	N/A	N/A	N/A	N/A	N/A	N/A
5	DCM	65	M	23	74	Y	N	Y	Y
6	DCM	39	F	17	80	Y	Y	Y	Y
7	DCM	41	M	9	84	Y	N	Y	Y
8	DCM	44	F	22	76	Y	Y	Y	Y

DCM, Dilated cardiomyopathy; LVEF, Left ventricular ejection fraction; LVEDD, Left ventricular end‐diastolic diameter; F, Female; M, Male; N/A, not available; Y, YES; N, NO.

### 2.3 Animal model

Procedures related to animals were in conformity with the National Institutes of Health Guide for the Care and Use of Laboratory Animals and received the approval of the Animal Care and Use Committees of the First Affiliated Hospital of Zhengzhou University. Male C57B/L6J mice were procured from the Institute of Laboratory Animal Science, Chinese Academy of Medical Sciences (Beijing, China). AAV9‐green fluorescent protein (GFP), AAV9‐TES, AAV9‐shTES, and scrambled shRNA were created. Mice that were 4 weeks old were injected with 100 μL of physiological serum solution containing 3 × 10^11^ genome copies per microlitre of AAV9 in the retro‐orbital sinus. AB was performed if hemodynamic stabilization was reached subsequent to the transduction of the virus.^25^, ^26^ Doppler assay was utilized to evaluate whether appropriate aortic constriction had been achieved. Mice that received sham operations were treated similarly but did not undergo aortic constriction. Echocardiography was applied to evaluate the wall thickness and the internal diameter of the LV at particular time points subsequent to the operation. Mice were sacrificed, and the hearts, tibiae, and lungs were obtained. Tissues were weighed and evaluated to determine the lung weight/body weight (LW/BW, mg/g), heart weight/tibia length (HW/TL, mg/mm), and HW/BW (mg/g) ratios of the groups.

### 2.4 Echocardiography and hemodynamic assessment

Echocardiography was conducted on mice anaesthetized with 1.5% isoflurane using a MyLab 30CV ultrasound machine (Biosound Esaote, Irvine, CA, USA) with a 10‐MHz linear‐array ultrasound transducer as described previously.^25^, ^26^, ^27^ The LV dimensions were evaluated in the parasternal short‐axis and long‐axis views with a frame rate of 120 Hz. The LV end‐diastolic diameter (LVEDD), ejection fraction (EF), LV end‐systolic diameter (LVESD), and fractional shortening (FS) were determined through M‐mode tracing with a sweeping velocity of 50 mm/s at the midpapillary muscle level.

For hemodynamic evaluation, a microtip catheter transducer (SPR‐839; Millar Instruments, Houston, TX, USA) was inserted into the right carotid artery and guided into the murine LV. Subsequent to a 15‐minute stabilization period, heart rate (HR) and pressure, and volume signals were measured with a Millar Pressure‐Volume System (MPVS‐400; Millar Instruments). Chart 5.0 software (Powerlab, AD Instruments, Shanghai, China) was utilized to analyse the results.

### 2.5 Histological examination

Hearts were resected and immediately placed in a ten percent potassium chloride solution to ensure that they were halted in diastole. A saline solution was used to wash the hearts, which were subsequently put into ten percent formalin. The hearts were transected near the apex to reveal the LVs and the right ventricles. Some slices with a thickness of 4‐5 μm underwent hematoxylin‐eosin (HE) staining in preparation for histopathology and picrosirius red (PSR) staining to enable evaluation of collagen deposition under a microscope. HE staining was used to examine the cross‐sectional area of cardiac muscle cells. A quantitative digital image analysis system (Image‐Pro Plus 6.0, Rockville, MD, USA) was used to examine the cardiomyocytes.

### 2.6 Immunofluorescent staining

Immunofluorescent staining was performed in tissue slices or neonatal rat cardiomyocytes (NRCMs) with a TES antibody to evaluate TES expression or with an a‐actinin antibody to evaluate cell surface area. Briefly, NRCMs underwent 24 hours of transduction with various adeno‐associated viruses and were subsequently stimulated for 48 hours with Ang II. Cells were fixed in 3.7% of formaldehyde in PBS before permeabilization with 0.1% of Triton X‐100 in PBS. Staining was performed with a 1:100 dilution of a‐actinin. The staining procedures were identical to those used for the NRCMs after the deparaffinization step.

### 2.7 Cardiomyocyte cultivation and transduction with recombinant adeno‐associated viral vectors

Cultivated NRCMs were treated as described previously, with minor changes.^25^, ^26^, ^27^ Briefly, PBS with 0.04% collagenase type II and 0.03% trypsin was used to separate cardiomyocytes from 1‐ to 2‐day‐old Sprague Dawley rats. After fibroblasts were eliminated with a preferential attachment technique, NRCMs were seeded in 6‐well plates (3 × 10^5^ cells/well) with a gelatin coating in DMEM/F12 that included twenty percent of fetal calf serum, penicillin/streptomycin, and BrdU (0.1 mmol/L, to suppress fibroblastic growth). The NRCMs subsequently underwent 12 hours of transduction with AdshTES and AdTES (MOI = 10). Media were replaced with serum‐free media for 12 hours before 48 hours of activation with one micromole of Ang II. The other procedures are described in the figure legends.

To overexpress TES, we used replication‐defective adeno‐associated viral vectors containing the entire rat TES cDNA coding region under the control of the cytomegalovirus promoter. An adeno‐associated viral vector that encoded GFP served as the control. To perform TES knockdown, three rat TES constructs were acquired from SABiosciences. We then created three Ad‐shTES adeno‐associated viruses and selected the one that caused noticeable endogenous TES downregulation for further experiments. Ad‐shRNA served as a non‐targeting control.

### 2.8 Western blotting

Heart tissues and cultivated cardiac myocytes were lysed in RIPA buffer. A BCA protein assay kit was used for protein quantification. Protein extracts (50 μg) were separated with SDS‐PAGE (Invitrogen, Carlsbad, CA, USA). The proteins were then transferred to a PVDF membrane (Millipore, Billerica, MA, USA) and incubated overnight with multiple primary antibodies at 4°C. After a 1 hour incubation with secondary antibodies at room temperature, the membranes were supplemented with ECL reagents (170‐5061; Bio‐Rad, Hercules, CA, USA) and observed with a FluorChem E imaging system (Cell Biosciences, Lake Franklin, NJ, USA). The expression of the proteins was normalized to that of GAPDH on an identical nitrocellulose membrane.

### 2.9 Reverse transcription‐PCR

Total mRNA was isolated from cultivated cells or LVs with TRIzol reagent (Invitrogen) and reverse transcribed into cDNA with a Transcriptor First Strand cDNA Synthesis Kit (Roche, Diagnostics, Mannheim, Germany). The relative expression of specific genes was evaluated using quantitative real‐time PCR with SYBR Green (Roche). The expression levels were normalized to GAPDH expression.

### 2.10 Calcineurin assay

The activity of calcineurin phosphatase was evaluated in cell extracts with a Calbiochem Calcineurin Cellular Activity Assay Kit (Ref. 207007). The calcineurin activity was evaluated by examining the free phosphate released with or without EGTA buffer. Colorimetric examination was performed with a plate reader (Dynatech MR 5000, Melville, NY, USA) at a wavelength of 620 nm.

### 2.11 Coimmunoprecipitation assays

For transient transduction and coimmunoprecipitation (Co‐IP) experiments, cultivated HEK293T cells were cotransduced for 48 hours with psicoR‐HA‐TES and psicoR‐Flag‐CaN and were then lysed in IP buffer (20 mmol/L Tris‐HCl, pH 8.0; 100 mmol/L NaCl; 1 mmol/L EDT; and 0.5% NP‐40 with added protease inhibitor cocktail) for 20 minutes. Centrifugation was used to eliminate precipitates. For every IP, 500 μL of specimen was incubated overnight with 10 μL of protein A/G agarose beads and 1 μg of antibody on a rocking platform. Finally, cold IP buffer was used to wash immunoprecipitates 5‐6 times prior to the addition of 1× loading buffer. The washed proteins were immunoblotted with specific primary antibodies.

### 2.12 Glutathione‐S‐transferase pull‐down assay

Glutathione‐S‐transferase (GST)‐fusion proteins containing truncated and full‐length calcineurin were expressed in Rosetta (DE3) *Escherichia coli*, purified, and immobilized on Glutathione Sepharose 4B beads (GE Healthcare, Beijing, China). Subsequently, the beads with bound proteins were incubated for 4 hours with HEK293T cell lysates with HA‐TES expression in IP buffer (20 mmol/L Tris‐HCl, pH 8.0; 150 mmol/L NaCl; 1 mmol/L EDTA; and 0.5% NP‐40 with added protease inhibitor cocktail) at 4°C. IP lysis buffer without protease inhibitor cocktail was used to wash the beads four times. Finally, the bound proteins on the beads were washed and resolved via SDS‐PAGE. Western blotting was carried out in preparation for analysis.

### 2.13 Luciferase reporter assay

A luciferase reporter assay was performed. Briefly, a lentivirus encoding NFAT bindings sites upstream of a firefly luciferase reporter gene was bought from QIAGEN (Lenti‐NFAT‐luc, CLS‐015L, Dusseldorf, Germany). Lenti‐NFAT‐luc accompanied by AdshTES or AdTES or a control virus were transduced into NRCMs for 24 hours. The cells were also activated for 24 hours with Ang II. Lysis buffer (Promega, Madison, WI, USA) was used to wash and lyse the harvested cells three times. After centrifugation, the supernatant was subjected to luciferase assay with a GloMax^®^ 20/20 Luminometer (Promega).

### 2.14 Statistical analysis

Data are presented as the means ± SEM. Unpaired Student's *t* tests and two‐way ANOVA with a Bonferroni posttest or a Tukey posttest were used to test for significant differences. *P* < 0.05 was recognized as significant. SPSS software (version 19.0; SPSS Inc., Chicago, IL, USA) was used to perform the statistical analyses.

## 3 RESULTS

### 3.1 Expression of TES is downregulated in human dilated cardiomyopathic hearts and murine hypertrophic hearts

**Figure 1:**
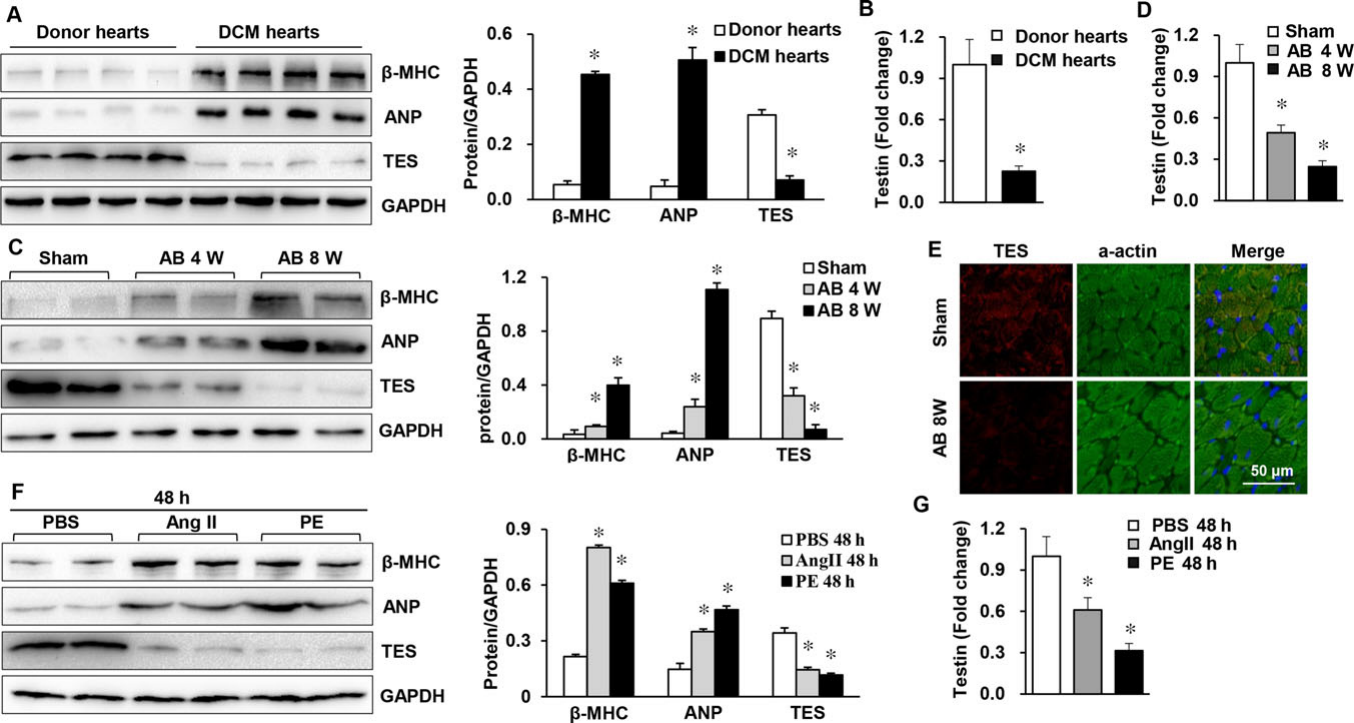
TES expression was suppressed in failing hearts and in murine cardiac hypertrophy models. A, Western blot analysis of markers of hypertrophy (ANP and β‐MHC) and TES protein levels in healthy donors and donors with dilated cardiomyopathy (n = 4 in each group). B, Real‐time PCR of testin in human hearts (n = 4 in each group, *P < 0.05 vs normal donor heart). C, Western blot analysis β‐MHC, TES, and ANP protein levels in hypertrophic hearts from mice receiving AB (n = 4 mice in each group). D, Real‐time PCR of testin in mouse hearts (n = 4 mice in each group, *P < 0.05 vs sham). E, Immunofluorescence staining of TES and a‐actin in mouse hearts (n = 5 mice in each group). F, Western blot analysis of β‐MHC, TES, and ANP in cultivated NRVMs triggered by Ang II (1 mol/L; n = 4) or phenylephrine (PE, 100 μmol/L) for 24 h. G, Real‐time PCR of testin in NRVMs in the indicated groups (*P < 0.05 vs PBS)

To explore the potential influence of TES on cardiac hypertrophy, our study evaluated whether TES expression was changed in diseased hearts. Human dilated cardiomyopathic samples were obtained, and it was discovered that TES mRNA and protein expression was remarkably suppressed, while β‐MHC and ANP (two biomarkers of hypertrophic hearts) expression was markedly increased, in comparison to the expression in healthy counterparts (Figure 1A and B). With regard to a murine model of cardiac hypertrophy triggered by AB, it was revealed that TES expression was noticeably suppressed in hypertrophic hearts after 4 or 8 weeks of AB in comparison to the expression in hearts that had received a sham operation (Figure 1C and D). In addition, TES was mainly localized to cardiomyocytes (Figure 1E). Moreover, it was discovered that TES mRNA and protein expression was clearly suppressed in hypertrophic cardiomyocytes in experiments with ex vivo cultivated NRCMs that received 48 hours of treatment with either phenylephrine (100 μmol/L) or Ang II (1 μmol/L) to trigger hypertrophy (Figure 1F and G). Overall, the above findings suggested that TES transcription and protein expression were noticeably suppressed in human dilated cardiomyopathic hearts, pressure overload‐triggered hypertrophic murine hearts, and ex vivo Ang II/phenylephrine‐treated cardiomyocytes, indicating that TES might participate in cardiac hypertrophy.

### 3.2 TES negatively regulates Ang II‐induced cardiomyocyte hypertrophy in vitro

**Figure 2:**
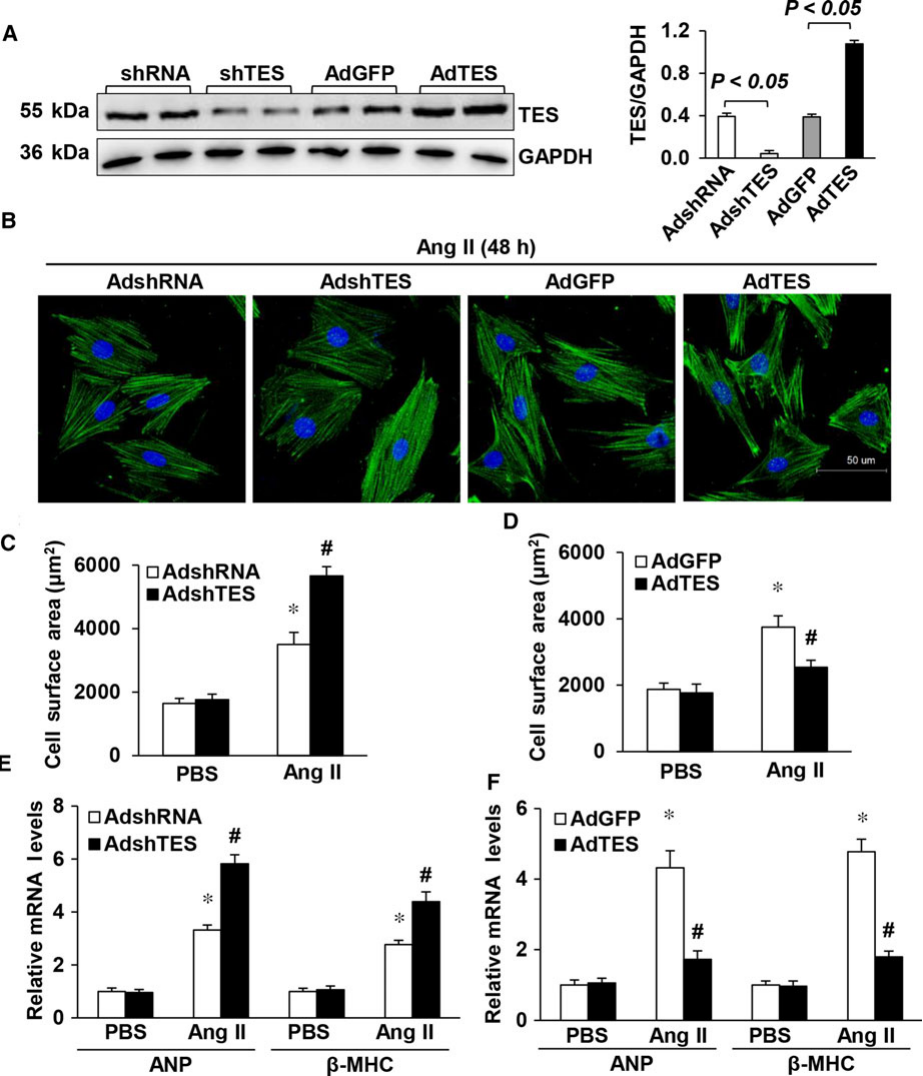
TES modulated Ang II‐triggered cardiac hypertrophy ex vivo. A, TES protein levels after transduction with AdshTES or AdTES (n = 3). B, Representative images of Ang II‐stimulated cardiac muscle cells that were transduced with AdTES or AdshTES. C, Quantification of cell surface area (n = 50 cells, *P < 0.05 vs AdshRNA/PBS; ^#^ *P* < 0.05 vs AdshRNA/Ang II). D, Quantification of cell surface area (n = 50 cells, *P < 0.05 vs AdGFP/PBS; ^#^ *P* < 0.05 vs AdGFP/Ang II). E, Real‐time PCR of β‐MHC and ANP in AdshTES cells and control AdshRNA cells after supplementation with PBS or Ang II for 48 h (n = 4, *P < 0.05 vs AdshRNA/PBS; ^#^ *P* < 0.05 vs AdshRNA/Ang II). F, Real‐time PCR of the mRNA concentration of β‐MHC and ANP in TES overexpressing cells and control (GFP) cells after supplementation with PBS or Ang II for 48 h (n = 4, *P < 0.05 vs AdGFP/PBS; ^#^ *P* < 0.05 vs AdGFP/Ang II)

To determine whether the changes in TES expression were in response to a hypertrophic irritant, our study examined whether TES was able to modulate the progression of cardiac hypertrophy by conducting loss‐of‐function and gain‐of‐function experiments in cultivated NRCMs. The TES concentration was suppressed via transduction of cardiomyocytes with AdshTES and was promoted via transduction with AdTES (Figure 2A). After transduction, cells were treated for 48 hours with either PBS (control) or Ang II (1 μmol/L) and were then immunostained with α‐actinin to measure the size of the cells. Noticeably, both knockdown (AdshTES) and excessive expression (AdTES) of TES failed to change the morphology or size of the cardiomyocytes under basal conditions (PBS) in comparison to the morphology and size after AdGFP or AdshRNA transduction. Nevertheless, in response to hypertrophy stimulation by Ang II, cells with a shortage of TES displayed noticeable increases in cell surface area (Figure 2B and C) in comparison to control cells, which were transduced with AdshRNA. In contrast, hypertrophy triggered by Ang II was noticeably inhibited in cardiomyocytes expressing TES in excess (Figure 2B and D). Accordingly, the mRNA expression of hypertrophy markers (β‐MHC and ANP) was clearly promoted in cardiomyocytes that were transduced with AdshTES (Figure 2E). However, the expression of these two markers was noticeably inhibited noticeably in cells transduced with AdTES (Figure 2F) in comparison to their expression in control cells. These findings confirmed that cardiac hypertrophy was suppressed by the upregulation of TES, while TES downregulation enhanced pathological cardiac hypertrophy.

### 3.3 TES overexpression suppresses AB‐induced cardiac hypertrophy

**Figure 3:**
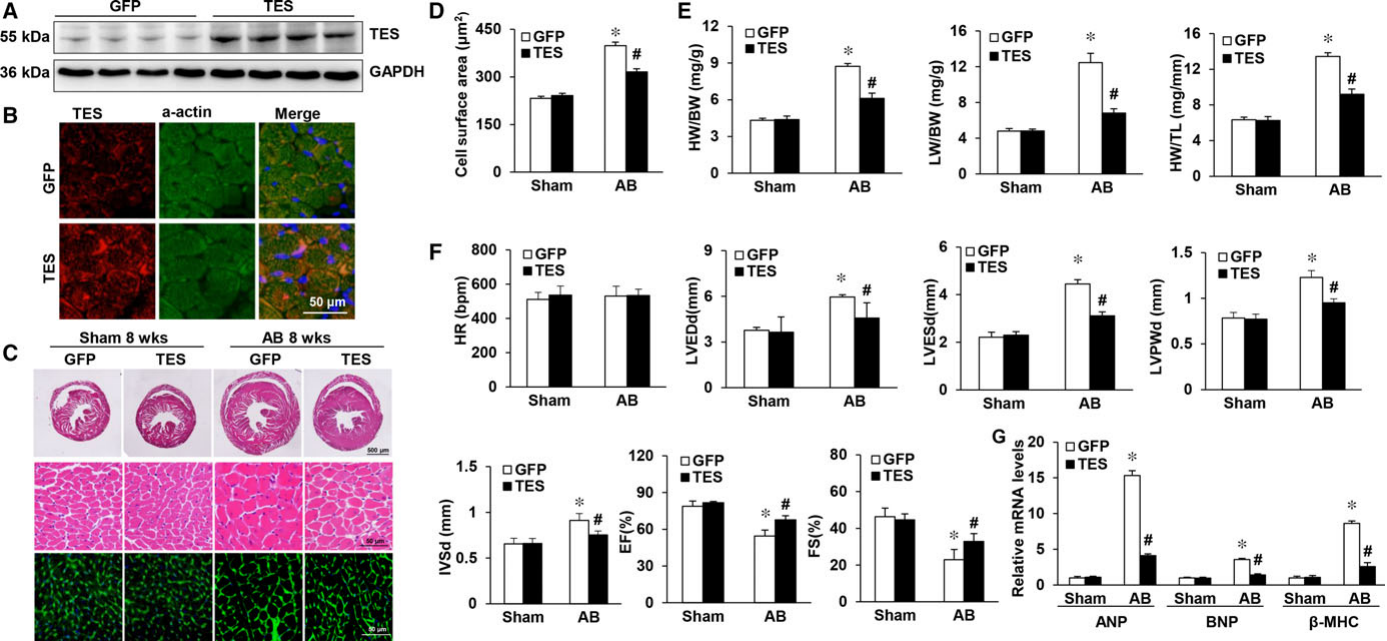
Overexpression of TES in the heart attenuates pressure overload‐induced hypertrophy. A, Western blot analysis was utilized to verify excessive TES expression (n = 4). GFP denotes the AVV9‐GFP groups, while TES denotes the groups with excessive expression of TES. B, Immunofluorescence staining of TES and a‐actin in mouse hearts after 8 weeks of AVV9‐GFP or AAV9‐TES injection (n = 4). C, Histological examination of HE staining and WGA staining of GFP and TES mice 8 weeks after the AB operation (n = 5‐6). D, Statistical analysis of the cardiomyocyte cross‐sectional areas (n = 100 cells). E, Statistical analysis of the LW/BW, HW/TL, and HW/BW ratios in the indicated groups (n = 11‐14). F, Echocardiographic parameters of TES as well as GFP mice (n = 9‐13). G, RT‐PCR of BNP, ANP, and β‐MHC expression triggered by AB in the indicated mice (n = 4). *P < 0.05 vs GFP/sham; ^#^ *P* < 0.05 vs GFP/AB

Our study subsequently explored whether elevated cardiac TES concentrations could inhibit the progression of cardiac hypertrophy and cardiac failure. A gene transfer approach using AAV9 was selected, as AAV9 exhibits a clear tropism for cardiomyocytes. Mice were injected with an AAV9 vector encoding TES or a control protein (GFP) in the retro‐orbital sinus. AB was conducted 4 weeks later. It was found via Western blot analysis that the expression of TES in hearts of AAV9‐TES mice was markedly elevated in comparison to that in control (AAV9‐GFP) hearts (Figure 3A and B). Importantly, the AAV9‐TES mice were fertile, viable, and displayed no pathological changes in cardiac morphology or activity at baseline. Unlike that of the AAV9‐GFP mice receiving AB treatment, the myocardial hypertrophic reaction was noticeably hindered in AAV9‐TES mice after 8 weeks of AB, which was revealed via direct evaluation of the gross heart, examination of HE and WGA‐fluorescein isothiocyanate staining, and evaluation of the cardiomyocyte cross‐sectional area (Figure 3C and D). The HW/TL, HW/BW LW/BW ratios were noticeably suppressed in AAV9‐TES mice in comparison to those in AAV9‐GFP mice after 8 weeks of AB (Figure 3E). It was suggested via echocardiography that these alterations enhanced cardiac dilation and hypertrophy and suppressed malfunction in AAV9‐TES mice compared to the corresponding parameters in AAV9‐GFP mice (Figure 3F). Additionally, AB promoted the mRNA levels of several hypertrophy markers, such as ANP, B‐type natriuretic peptide (BNP) and β‐MHC, in hearts of AAV9‐GFP mice, and the expression of the markers was noticeably inhibited in AAV9‐TES mice (Figure 3G). Overall, the above findings suggested that excessive TES expression was able to inhibit the development of cardiac hypertrophy triggered by AB in vivo.

### 3.4 TES overexpression attenuated fibrosis in pressure‐overloaded hearts

**Figure 4:**
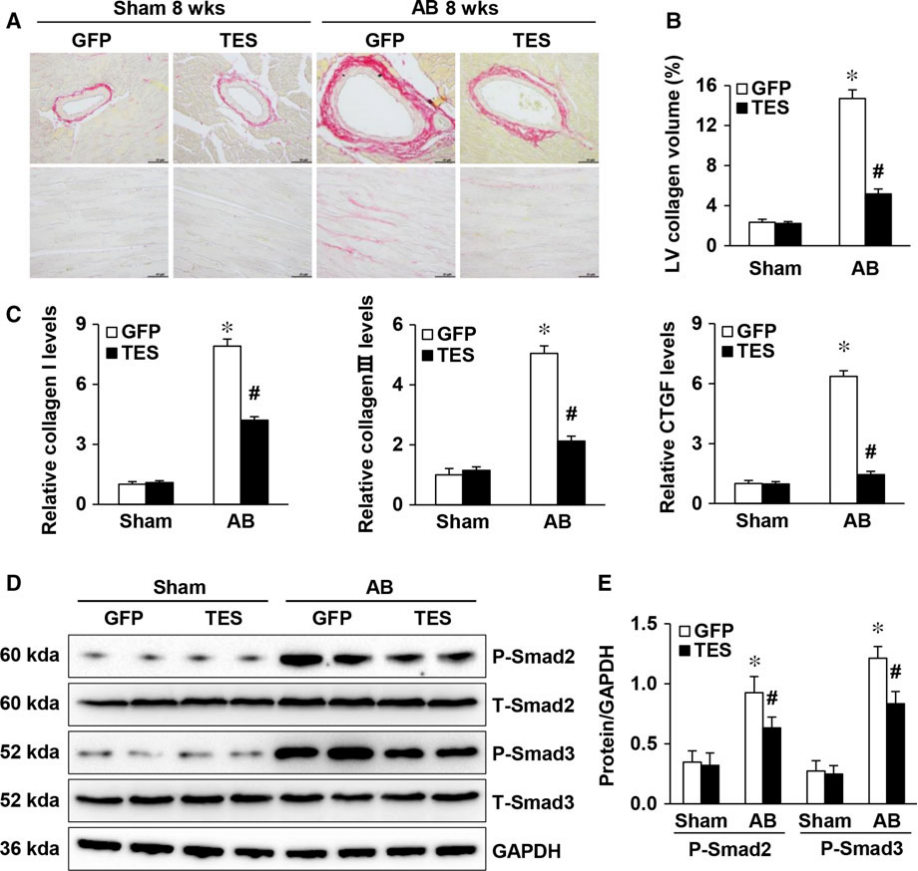
TES overexpression attenuated fibrosis in pressure‐overloaded hearts. A, Picrosirius red staining of histological slices of LVs in the indicated groups 8 weeks after AB (n = 6, scale bar = 50 μm). B, An image analysis system was used to quantify the fibrotic area (n = 29‐33 fields). C, qPCR of markers of fibrosis (collagen I, CTGF, and collagen III) in the indicated mice (n = 4). D and E, Representative Western blot and quantification of total and phosphorylated Smad 2 and 3 proteins in the indicated groups (n = 4).*P < 0.05 vs GFP/sham; ^#^ *P* < 0.05 vs GFP/AB

Cardiac hypertrophy features fibrosis, which is manifested by cardiac collagen deposition. To examine the influence of excessive TES expression on the maladaptive remodelling of hearts, this study examined the participation of TES in fibrosis. The severity of fibrosis was quantified based on the volume of collagen through the visualization of the total quantity of collagen in the interstitial and perivascular spaces. Our study revealed that interstitial and perivascular fibrosis was noticeably reinforced in AAV9‐GFP hearts that received chronic AB. However, such fibrosis was markedly inhibited in AAV9‐TES hearts (Figure 4A and B). Next, we examined collagen generation by evaluating the transcription of fibrotic markers such as collagen I, connective tissue growth factor (CTGF) and III. It was revealed that the fibrotic reaction was impaired in AAV9‐TES mice in comparison to that in AAV9‐GFP mice (Figure 4C). The TGF‐β/Smad pathway is an essential pathway participating in the progression of cardiac fibrosis. To evaluate the role of TES in the etiology of collagen generation, our study explored the modulation of Smad cascade stimulation by TES. It was revealed that the enhanced Smad 2/3 phosphorylation triggered by AB was noticeably suppressed in AAV9‐TES mouse hearts in comparison to that in AAV9‐GFP hearts (Figure 4D and E). In summary, the above findings suggested that excessive TES expression was able to impair myocardial fibrosis triggered by pressure overload.

### 3.5 TES inhibited the calcineurin‐NFAT pathway in murine hypertrophic hearts

**Figure 5:**
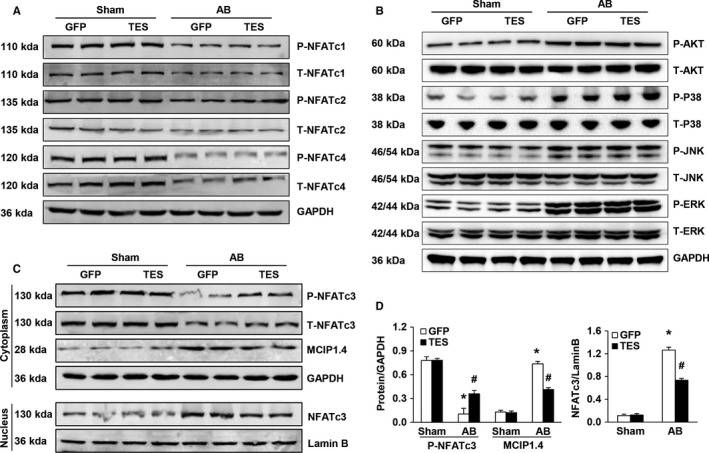
TES inhibited the calcineurin‐NFAT axis in murine hypertrophic hearts. A, Western blot of the total and phosphorylated protein concentrations of NFATc4, NFATc2, and NFATc1 in murine hearts in the indicated groups. B, Western blot of the total and phosphorylated protein concentration of MEK1/2, AKT, ERK1/2, P38, and JNK1/2 in murine hearts in the indicated groups. C and D, Representative Western blot and quantification of NFATc3 and MCIP1.4 protein levels in murine cytoplasm and nucleus in the indicated groups. (n = 4, *P < 0.05 vs GFP/sham; ^#^ *P* < 0.05 vs GFP/AB)

This study next explored the mechanisms by which TES represses hypertrophy. Previous research has proven that the calcineurin‐NFAT pathway participates in cardiac hypertrophy and that the muscle LIM protein is required for calcineurin‐NFAT signalling at the sarcomeric Z disc.^28^, ^29^ Consequently, our study examined whether TES modulates cardiac hypertrophy via the calcineurin‐NFAT axis. It has previously been proven that stimulated calcineurin directly links with NFAT transcription factors, bringing about nuclear translocation and dephosphorylation of NFAT. Thus, our study evaluated cardiac NFAT translation triggered by pressure overload. It was discovered that the AB‐mediated decrease in p‐NFATc3 expression in the cytoplasm was noticeably promoted in the AVV9‐TES group in comparison to that in the AVV9‐GFP group. However, our study failed to observe any differences in p‐NFATc1, p‐NFATc2, and p‐NFATc4 between the groups (Figure 5A, C and D). Despite the fact that the AKT pathway and the MAPK pathway both modulate cardiac remodelling, our study failed to detect any differences in AKT or MAPK stimulation between the groups (Figure 5B). Overall, the above findings indicated that TES‐modulated pathological cardiac hypertrophy relied, at least in part, on the modulation of the calcineurin‐NFAT axis.

### 3.6 TES directly interacts with calcineurin

**Figure 6:**
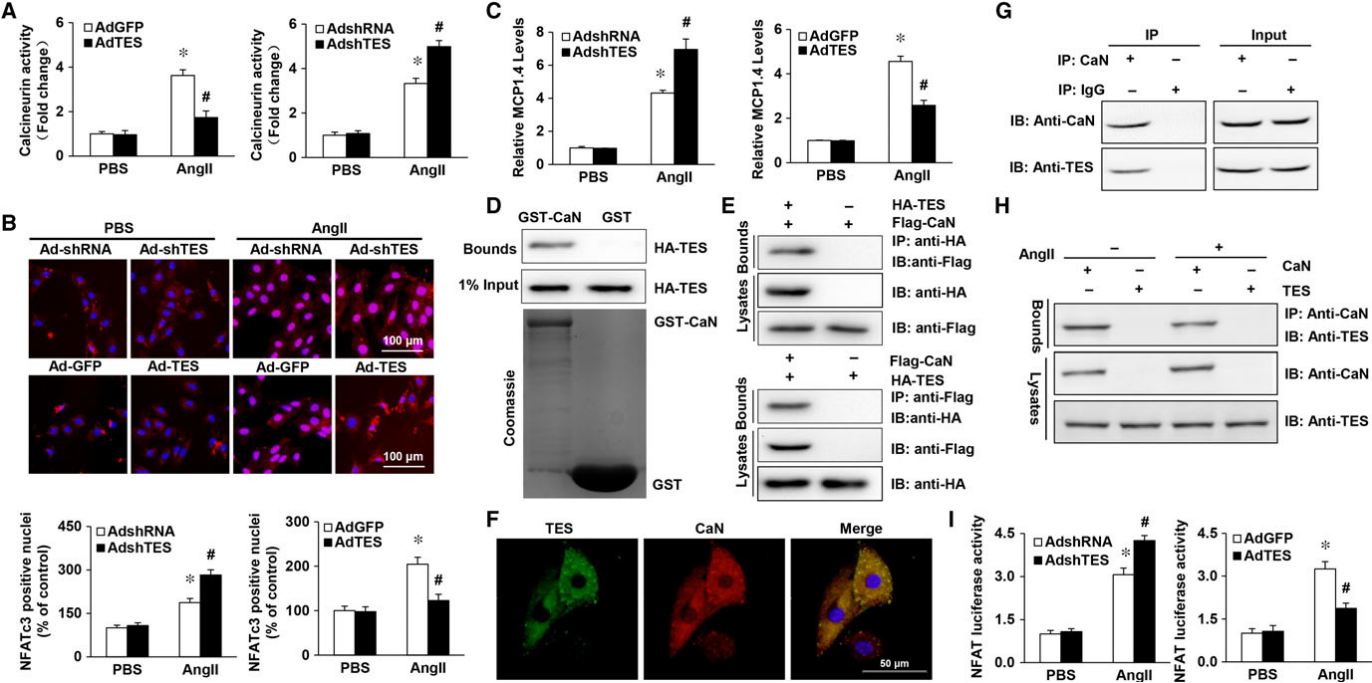
Mechanism of TES modulation of the calcineurin‐NFAT Axis. A, The calcineurin activity of NRVMs that were transduced with the indicated plasmids and stimulated by AngII for 30 min was evaluated. B, Immunofluorescence staining and quantitative analysis of the nuclear translocation of NFAT (n = 100 cells). C, Real‐time PCR analysis of the 1.4 isoform of the calcineurin‐interacting protein (MCIP 1.4) in mouse hearts from the indicated experimental groups (n = 6). D, A GST pull‐down assay was performed to examine the direct interaction between calcineurin and TES. E, Co‐IP assays proving the existence of an interaction between calcineurin and TES in vitro. F, Colocalization of TES and calcineurin in the cytoplasm. G, Co‐IP assays proving the existence of an interaction between calcineurin and TES in vivo. H, Co‐IP assays proving the existence of an interaction between calcineurin and TES in H9c2 cells stimulated with AngII. I, Relative luciferase activity of the NFAT promoter in NRVMs cotransduction with adeno‐associated viruses

Subsequently, we analysed the mechanism by which TES regulates calcineurin activity. In this study, we proved that NFAT nuclear translocation and calcineurin stimulation triggered by AngII were noticeably inhibited in NRVMs that were transduced with AdTES in comparison to the NFAT translocation and calcineurin stimulation in controls. In contrast, AdshTES reinforced the calcineurin stimulation and NFAT nuclear translocation triggered by AngII, suggesting that the phosphatase is a target of TES (Figure 6A and B). In agreement with this finding, the expression of the 1.4 isoform of the calcineurin‐interacting protein (MCIP 1.4), which acts as a downstream effector of functions relying on calcineurin as well as a highly sensitive marker for calcineurin function,^30^, ^31^ was reduced in the AdTES‐supplemented group triggered by AngII in comparison to the expression in the control group. However, the AdshTES group displayed the opposite effect (Figure 6C). It has previously been proven that LIM domains participate in interactions between proteins. MLP and FHL2 are both able to interact with calcineurin,^24^, ^28^ which raises doubt as to whether TES can act directly on the function or structure of calcineurin. Consequently, our study aimed to explore the potential interactions between calcineurin and TES. A GST pull‐down assay was performed to examine whether TES directly interacts with calcineurin. It was demonstrated that TES bound to calcineurin (Figure 6D). This interaction was further confirmed with a Co‐IP assay (Figure 6E). Moreover, an interaction between TES and CaN in vivo was detected. Our results showed that TES could interact with CaN in vivo (Figure 6G). Then, we detected an interaction between TES and CaN after cells were treated with AngII. The results showed that the interaction between TES and CaN was attenuated when H9c2 cells were treated with AngII (Figure 6H).

Furthermore, our study investigated the localization of calcineurin and TES using fluorescent confocal microscopy. It was discovered that both calcineurin and TES were mainly located in the cytoplasm and fused rather well (Figure 6F). The interaction between the calcineurin‐NFAT axis and TES was verified via cotransduction of AdTES or AdshTES with NFAT reaction elements conjugated to a luciferase gene (NFAT‐Luc) in NRVMs. It was discovered that NFAT‐Luc transcription triggered by Ang II was noticeably promoted in cells that underwent transduction with AdshTES in comparison to the AngII‐triggered transcription in control AdshRNA cells. In contrast, the promotion of NFAT‐Luc transcription triggered by Ang II was markedly suppressed in AdTES cardiomyocytes (Figure 6I). Overall, the above findings indicated that TES inhibited the NFAT pathway via interaction with the stimulated calcineurin pathway.

### 3.7 Blocking calcineurin‐NFAT signaling blunts cardiac hypertrophy in TES knockdown mice

**Figure 7:**
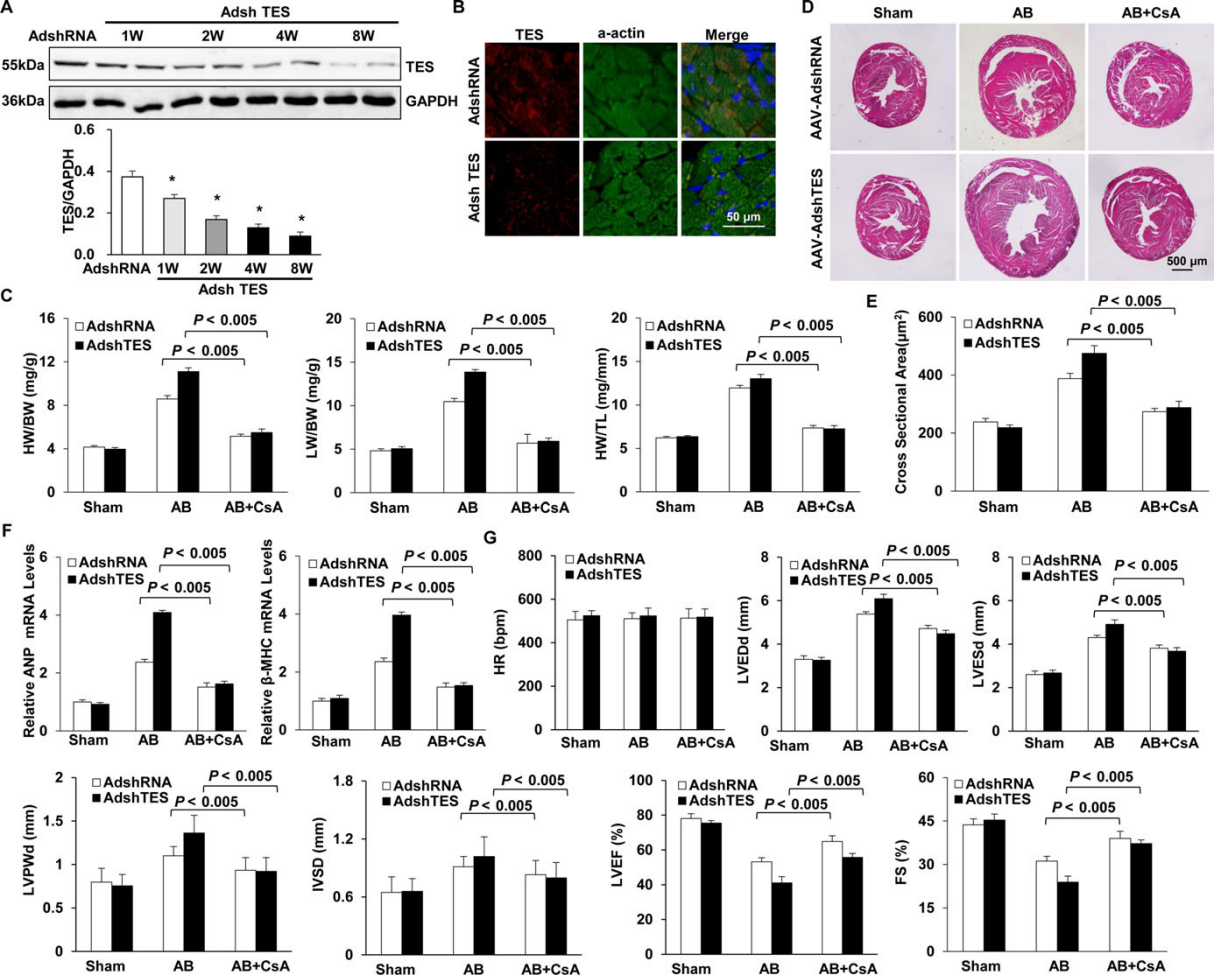
Blocking calcineurin‐NFAT signalling blunts cardiac hypertrophy in TES knockdown mice. A, TES protein expression level in mouse hearts after 1, 2, 4, or 8 weeks of AAV9‐shTES injection (n = 4, *P < 0.05 vs AdshRNA group). B, Immunofluorescence staining of TES and a‐actin in mouse hearts after 8 weeks of AAV9‐shRNA or AAV9‐shTES injection (n = 4). C, Statistical analysis of the ratios of LW/BW, HW/TL as well as HW/BW in indicated groups (n = 11‐15). D, HE staining and WGA staining in mice 4 weeks subsequent to AB operation in indicated groups (n = 9‐11). E, Statistical analysis for cross‐sectional area (n = 100 cells). F, RT‐PCR of β‐MHC and ANP triggered via AB in indicated mice (n = 4). G, Echocardiographic parameters in indicated groups (n = 9‐11)

To better verify the essential influence of the calcineurin‐NFAT axis in vivo, the calcineurin suppressor CsA was used. Mice received an injection of an AAV9 encoding shTES or the control (shRNA) in the retro‐orbital sinus (Figure 7A and B). AB was conducted 4 weeks later. The mice were given CsA (25 mg/kg/day) supplements or vehicle 1 week prior to the operation. The treatment lasted for 4 weeks after the AB or sham operation. AAV9‐shTES mice, which received vehicle, displayed obvious hypertrophy based on the cardiomyocyte cross‐sectional areas and the HW/BW, HW/TL, and LW/BW ratios (Figure 7C‐E) in comparison to their AAV9‐shRNA counterparts 4 weeks after AB. These alterations were noticeably attenuated in CsA‐supplemented AB AAV9‐shTES mice. Furthermore, the expression of markers of hypertrophy (β‐MHC and ANP) triggered by AB was noticeably inhibited by preliminary CsA supplement in AAV9‐shTES mice (Figure 7F). The activity and structure of the heart was noticeably promoted in CsA‐supplemented AB AAV9‐shTES mice as indicated by echocardiographic parameters (Figure 7G). In general, the findings of our study suggested that CsA prevented cardiac hypertrophy development in TES knockdown mice by hindering the calcineurin‐NFAT axis. Our findings also suggested that the protective influence of TES against cardiac hypertrophy was related to the modulation of the calcineurin‐NFAT axis.

## 4 DISCUSSION

Cardiac hypertrophy is a common reaction to multiple pathological irritants and ultimately causes heart failure, underlining the importance of identifying reliable targets through which to inhibit its progression. Our study proved that TES expression was inhibited in failing human heart specimens as well as in murine cardiac hypertrophy models triggered by pressure overload. Loss and gain of function modulated by an adeno‐associated virus suggested that suppressed TES expression enhanced the Ang II‐triggered hypertrophy of cardiac muscle cells in vitro, while TES recovery noticeably impaired the influence of Ang II. To better verify the cardiac influence of TES in models in vivo, we generated mice with excessive TES expression specific to the heart by using an adeno‐associated virus expression system. Excessive TES expression in hearts defended against cardiac hypertrophy and fibrosis triggered by pressure overload, which was consistent with the in vitro findings. The influence of TES on cardiac hypertrophy mainly relied on a blockade of the calcineurin‐NFAT axis. Thus, our study is the first to determine the influence of TES on heart and to suggest that TES is a potential target for the treatment of pathological cardiac hypertrophy.

Human TES is located in the cytoplasm and is a constituent of focal adhesions and a participant in interactions among cells. Human TES consists of a PET domain (discovered in a few proteins whose activity remains unknown) and three tandemly arranged LIM domains (protein interaction motifs that bind various distinct proteins).^6^, ^7^ It is widely accepted that LIM domain proteins are essential modulators of cell proliferation, fate determination, differentiation, and the remodelling of the cytoskeleton.^17^, ^18^, ^19^ Increasing proof has suggested that proteins with LIM domains are crucial in pathological conditions in hearts including cardiac hypertrophy, ischemic injury, and heart failure. Recent studies have proven that Lmcd1 participates in the development of cardiac hypertrophy.^23^ Hojayev and his colleagues proved that FHL2 was able to inhibit the pathological proliferation of the heart.^28^ The above findings reliably indicate that TES is an essential modulatory of cardiac hypertrophy. However, current understanding of the modulation of TES expression, particularly during the transition from cardiac hypertrophy to cardiac failure, is insufficient. Our study is the first to prove that TES is a reverse modulator of cardiac hypertrophy in cardiomyocytes, and it did so with an adeno‐associated virus expression system. Nevertheless, further research is still needed elucidate the mechanism by which TES acts on the heart.

Multiple signalling pathways that include transcription factors, kinases, and G‐protein coupled receptors participate in the modulation of heart growth. It has been proven that the calcineurin‐NFAT axis is essential in regulating cardiac hypertrophy.^28^, ^29^ NFATs are dephosphorylated by calcineurin in response to elevated intracellular calcium. Calcineurin modulates the expression of genes in various tissues sensitive to calcium, including muscle, brain, and lymphocytes. NFAT transcription relies on dephosphorylation by calcineurin, which brings about nuclear translocation and the stimulation of targeted genes.^32^, ^33^ It has recently been proven that the LIM protein in muscles is directly related to calcineurin and is crucial to the calcineurin‐NFAT axis.^23^, ^28^ However, little research has been conducted on the direct influence of TES on the calcineurin‐NFAT axis. Our study is the first to prove that TES upregulation inhibits the calcineurin‐NFAT axis in cardiac hypertrophy. Furthermore, GST pull‐down assays, fluorescent confocal microscopy and Co‐IP assays were performed to examine whether there was an interaction between TES and calcineurin. It was discovered that TES was able to directly interact with calcineurin. Additionally, the findings above proved that excessive TES expression hindered the promotion of MCIP1.4 and the stimulation of NFAT promoters in NRVMs triggered by AngII. However, TES knockdown in NRVMs promoted the elevation of MCIP1.4 and the stimulation of NFAT promoters. Moreover, pharmacological suppression of the calcineurin pathway with CsA supplementation in TES knockdown mice impaired cardiac hypertrophy. These findings indicate that the calcineurin‐NFAT axis is a crucial contributor to the counteracting effect of TES on cardiac hypertrophy.

In all, our study revealed a novel role for TES in the modulation of pathological cardiac hypertrophy through suppression of the calcineurin‐NFAT axis. Our research is the first of its kind to prove the participation of TES in cardiac hypertrophy and to suggest that proteins consisting of LIM domains can act as a reservoir of signalling agents related to the preservation of cardiac homeostasis. We have discovered in this study that TES defends the heart in response to pressure overload, indicating that TES can serve as a promising diagnostic and therapeutic target for cardiac illness. However, further verification of the influence of TES on cardiac hypertrophy is necessary.

## 5 CLINICAL PERSPECTIVES

This research offers in vitro and in vivo proof that excessive TES expression attenuates cardiac hypertrophy through suppression of the calcineurin‐NFAT axis. We have verified that TES has an influence on cardiac hypertrophy in response to pressure overload and that TES is related to the calcineurin‐NFAT axis in cardiac hypertrophy. The above findings are essential to elucidate the etiology of cardiac hypertrophy and to develop innovative strategies to treat cardiac hypertrophy by targeting TES.

## CONFLICT OF INTEREST

The authors declare no conflicts of interest.
